# Complexity in African savannas: Direct, indirect, and cascading effects of animal densities, rainfall and vegetation availability

**DOI:** 10.1371/journal.pone.0197149

**Published:** 2018-05-16

**Authors:** Tim Leeuwis, Mike Peel, Willem F. de Boer

**Affiliations:** 1 Resource Ecology Group, Wageningen University, Droevendaalsesteeg 3a, Wageningen, The Netherlands; 2 Agricultural Research Council, Animal Production Institute, Nelspruit, South Africa; Indian Institute of Science, INDIA

## Abstract

Savanna ecosystems are popular subjects for interaction studies. Multiple studies have been done on the impact of elephants on vegetation, the impact of grass and browse availability on animal densities or on competition between herbivore species. Previous studies showed that elephant densities are frequently negatively correlated with densities of tall trees, and that browse and grass availability are correlated with browser and grazer density respectively. Additionally, a competition effect between browse and grass availability has been reported. These relationships are usually analysed by testing direct relationships between e.g., herbivore densities and food availability, without addressing competition effects or other indirect effects. In this study, multiple interactions in a savanna system have been analysed simultaneously using Partial Least Square-Path Modelling (PLS-PM) using mammal and vegetation data from three different wildlife reserves in southern KwaZulu-Natal. The results showed that the processes that three separate models for the three areas provided the best understanding of the importance of the different interactions. These models suggest that elephants had a negative impact on trees, but also on grass availability. The impact is stronger when elephants are not able to migrate during the dry season. Browsers and grazers were correlated with browse and grass availability, but competition between browse and grass was not detected. This study shows that due to the complexity of the interactions in an ecosystem and differences in environmental factors, these interactions are best studied per area. PLS-PM can be a useful tool for estimating direct, indirect, and cascading effects of changing animal densities in conservation areas.

## Introduction

In a savanna ecosystem, there are many factors that vary over the years, such as rainfall, animal numbers, and vegetation biomass and composition [1]. These factors, together with their interactions, play an important role in determining the composition and structure of the savanna ecosystem. Changes in one variable, influencing another variable (i.e. direct effects) can trigger far reaching, cascading effects [2]. However, many studies only focussed on simple one-to-one relationships in their analysis, such the correlation between vegetation biomass and herbivore densities [3–5], or the effect of rainfall on vegetation biomass [4], while ignoring the direct and indirect effects of other variables in the system, such as competitive and facilitative effects that, together, structure the vegetation and herbivore community composition. Hence, in this study, a PLS-PM is used that is able to address this analysis bias, quantifying the direct and indirect effects of multiple variables in a single approximate analysis.

Vegetation biomass is influenced by multiple factors, but is primarily regulated by moisture availability from rainfall and groundwater [6]. However, grass is much more dependent on rainfall than trees, because tree roots reach further and can make use of deeper water layers that grass roots cannot reach [7]. Hence, a drought of a year can have a heavy impact on the grass availability while the browse availability is less affected [7], and this interaction is expected to also influence the grazer and browser densities in savannas.

Moreover, many herbivore species are known to be bottom-up regulated, especially the larger herbivore species [8]. This means that there can be competition for browse and grass between herbivores if food availability is limiting [9]. Plant species also interact, as trees and grasses compete for limiting water and nutrient resources. The competition between trees and grass for water is probably not large because of niche differentiation, but many tall trees increase the canopy cover, so that sunlight does not reach the grass layer [7]. If trees are uprooted or heavily browsed, the landscape becomes more open, which can have a positive influence on the grass layer, triggering a change in species composition of the vegetation [5].

Grazers are affected by grass availability, browsers by browse availability and mixed feeders by both. Mixed feeders are capable of changing their diet composition when the availability of either browse or grass is low (Kos et al. 2012). When there is enough grass of sufficient high quality, the mixed feeding impala (*Aepyceros melampus*) will prefer to feed on grass, but if the grass availability becomes limiting, impala shift to browse [8,10]. If the browse:grass ratio changes, the herbivore community composition is expected to follow the changes in grass and browse availability (de Boer et al. 2015). Elephants (*Loxodonta africana*) are also mixed feeders, feeding both on grass and on browse. Smaller trees can be easily browsed or even consumed whole. Larger trees, with fresh leaves outside their reach, are debarked, pushed over or uprooted in order to browse on the higher, fresher leaves and roots. This browsing behaviour influences the tree density and the tree height class distribution [11]. However, elephants can also feed on grasses and reduce total grass biomass [3], and therefore have a large competitive effect on smaller herbivore species, especially in areas where there is high annual rainfall and low soil nutrients, because smaller ungulates need higher quality browse and grass [4,12]. Hence, negative effects have been found with increasing elephant densities on different herbivore species [13]. However, due to the uprooting of trees and the large quantities of browse being consumed by elephants, elephants might also compete with large browsing herbivores such as giraffes (*Giraffa camelopardalis*) [4].

Apart from the competitive effects of elephants on other herbivores, facilitative effects have also been reported. The most discussed effect is the increase in browse availability at lower heights, because of the uprooting and hedging of trees [14]. It is also shown that because of elephant impact, regrowth of browse is stimulated in the dry season, improving browse quality [15]. Elephants could have a positive effect on tree cover and biomass, as small trees regrow fast at places where elephants have killed larger trees with an associated shift from larger to smaller trees [16,17], increasing the accessible browse biomass, and thereby facilitating smaller browsers [18,19]. These effects are mainly relevant for smaller browsing species, but there is also a positive effect of elephant densities on grass regrowth [3,20,21]. As a net effect of both competitive and facilitative effects the herbivore community composition may shift to a grazer and megaherbivore dominated community with increasing elephant densities [11].

In short, many different interactions have been reported with both direct and indirect, cascading effects. These interactions have usually been analysed using separate one-to-one relationships, which makes it difficult to determine the exact causality of the shown effects. For example when elephants have a negative effect on grass, and grass has a positive effect on grazers, there is an indirect negative effect of elephants on grazers. However, the negative effect of elephants on trees and the competition effect between trees and grass should also be included in the analysis for a better understanding on increasing elephant densities, so therefore it becomes hard to draw a conclusion about the net effect of elephants on the system. The most important interactions in a savanna ecosystem have therefore, in this study, all been investigated with a single analysis, using Partial Least Square-Path Modelling (PLS-PM; [22]). PLS-PM creates an interaction diagram, and all interactions are analyzed, while taking each other’s direct and indirect influences into account, providing better insight into the different interactions in the ecosystem. It should however be noted that even though PLS-PM provides a good representation of these interactions, it is still an approximate method and, similarly to correlative analyses, is not able to test for causal effects.

The expectations are that rainfall is stronger correlated with grass availability than with trees, because trees can use deeper ground water. Moreover, elephant density is expected to be stronger correlated with densities of tall trees than of small trees and grass, because of the uprooting and debarking of large trees by elephants. A strong correlation is expected between browse availability and browsers and between grass availability and grazers. For the mixed feeders a stronger correlation is expected with grass availability than with browse availability, since grass is their first choice [8,10]. Lastly, a competition effect between browse and grass is expected, in which tall trees have a smaller influence than small trees due to niche differentiation. With the results of this research, a better insight on the impacts of elephant can be obtained, allowing reserve managers to better understand the direct and indirect effects of, for example, a growing elephant population on their conservation area.

## Materials and methods

### Study sites

All data were collected in three protected areas (Table 1) in southern KwaZulu-Natal, adjacent to Kruger National Park. All three areas had an extensive and spatial dataset, with varying numbers of elephants and with varying environmental factors. Kapama Game Reserve (13000 ha) is a private game reserve which is completely fenced. The Klaserie River runs through the reserve as a natural water source. Balule Game Reserve is also a private game reserve, and is open to the Kruger National Park. This reserve is a collaboration of multiple smaller privately owned parks, all with open borders to each other, which together covers 40000 hectares. Overall, the reserve has less rainfall and a weaker grass layer. The Olifants River runs through the reserve as a natural water source. Sabi Sands Wildtuin (65000ha) is the largest reserve of the three. It is open to Kruger National Park, lies much further to the south than the other reserves, and has a higher rainfall. The Sabi and Sand Rivers run through the reserve. All three reserves have multiple artificial waterholes.

**Table 1:** Overview of characteristics of the different research areas, with the name and size of the reserve, the average rainfall per year, the number of years from which complete data is available for analysis, and whether the reserve has open borders to Kruger National Park.

Reserve		Size (ha)	Average rainfall (mm)	Years of data available	Period of data collection	Open borders to Kruger NP
Balule Game Reserve		40000	477	15	1999–2014	✓
Kapama Game Reserve		13000	565	20	1995–2014	X
Sabi Sand Wildtuin		65000	665	22	1993–2014	✓

### Data analysis

Data on mammals and vegetation were analysed using R-statistics 2.13.1 (R Development Core Team 2011), using Partial Least Square-Path Modelling (PLS-PM; [22]), which calculates all different relationships in an interaction diagram and quantifies the influence of each variable on the other by calculating regression coefficients. PLS-PM uses the construct of an inner model and an outer model. This inner model includes the latent variables and how they influence each other. This is the actual interaction diagram which was used to analyse the data. These latent variables are variables that have not been measured directly in the field, but are described by one or more actual measured variables, called manifest variables, which are part of the outer model. There is one important downside to PLS-PM, namely that the analysis does not allow loops in the system, so for example the competition effect between grass and trees can only be implemented in the analysis in one direction (for example impact of trees on grass). The loops were therefore replaced by one-sided effects, by carrying out the analysis twice, with both ways to calculate which effect was stronger, and the strongest relationship was used in the final model.

When constructing a path model, first an interaction diagram was created to function as the inner model. In order to make a realistic interaction diagram, a linear regression analysis between all different variables was done to quantify the correlation between two variables in the system. With the outcomes from these analyses a realistic interaction diagram could be constructed. Single species have been used instead of pooled samples of all grazers, browsers and mixed feeders, since the collected data was more precise for some species than for others. For grazers, zebra density was used, for browsers, giraffe density was used, and for mixed feeders, impala density was used.

Another important aspect for constructing the inner model is the lag effect. This means that some interactions only take effect after one or more years and not directly. For example more rainfall in a particular year may result in more trees in the next year, and will result in a stronger correlation if the rainfall from this year is compared to the tree density of the next year. Therefore, all correlation tests were carried out several times, with a zero, one or two year lag effect for all variables where such a lag effect could be expected, and the strongest correlation was used for the inner model.

All data were available from the Agricultural Research Council (ARC) in Nelspruit as part of the annual monitoring surveys; all data points are averages estimates of multiple measurements per year (Table 2, S1 Table). For the grass data collection, a transect was set out over a total length of 100 m in a square (4 x 25 m) every year at the same site. The measurements were taken along this transect every year in the same way. To measure the grass standing crop (kg/km^2^), a disc pasture meter was used, the grass biomass was sampled 33 times along the transect (once every 3 m). At every meter mark in the transect, the closest grass species (annual or perennial) was identified and the distance to the centre of the tuft was measured. If this grass was an annual, the closest perennial was also identified and the distance to the centre of the tuft was measured. Also, the diameter of the measured grass tufts was measured in millimetres. With these measurements, the perennial percentage (%), the distance to perennial (mm), and the tuft size (mm) were calculated.

**Table 2:** All measured manifest variables used in the analyses with abbreviations and dimensions.

Manifest variable	Abbreviation	Dimension
Rainfall per year	rainfall	mm/year
Tree density	density	#stems/transect
Height class 1–4 trees	hc1-4	#stems/transect
Tree cover	canopy	%
Perennial percentage	peran	%
Grass standing crop (biomass)	SC	kg/ha
Distance to closest perennial	dis.per	Mm
Tuft diameter	tuft	Mm
Elephant numbers	eleph#	#/km^2^
Zebra numbers	zebra#	#/km^2^
Impala numbers	impa#	#/km^2^
Giraffe numbers	gira#	#/km^2^

For the tree data collection, the same transect was used as for the grass data collection. Along this transect, trees were identified within a 2m belt and the height class was recorded for every tree in 4 height classes: 1 = 0–1 m, 2 = 1.1–2 m, 3 = 2.1–5 m; 4 = > 5 m. To measure the density (#stems/transect), the total number of stems measured at a transect was recorded. To measure canopy cover (%), at every meter point a vertical projection was made to determine the percentage of the transect that was covered by woody canopy.

The animal numbers were obtained from annual aerial surveys, where a helicopter flew the same transect every year across the entire reserve and the same team counted all animals. This gives an indication of the changes in animal numbers in a reserve over the years. Fire data were excluded as the data set was incomplete.

**Fig 1:**
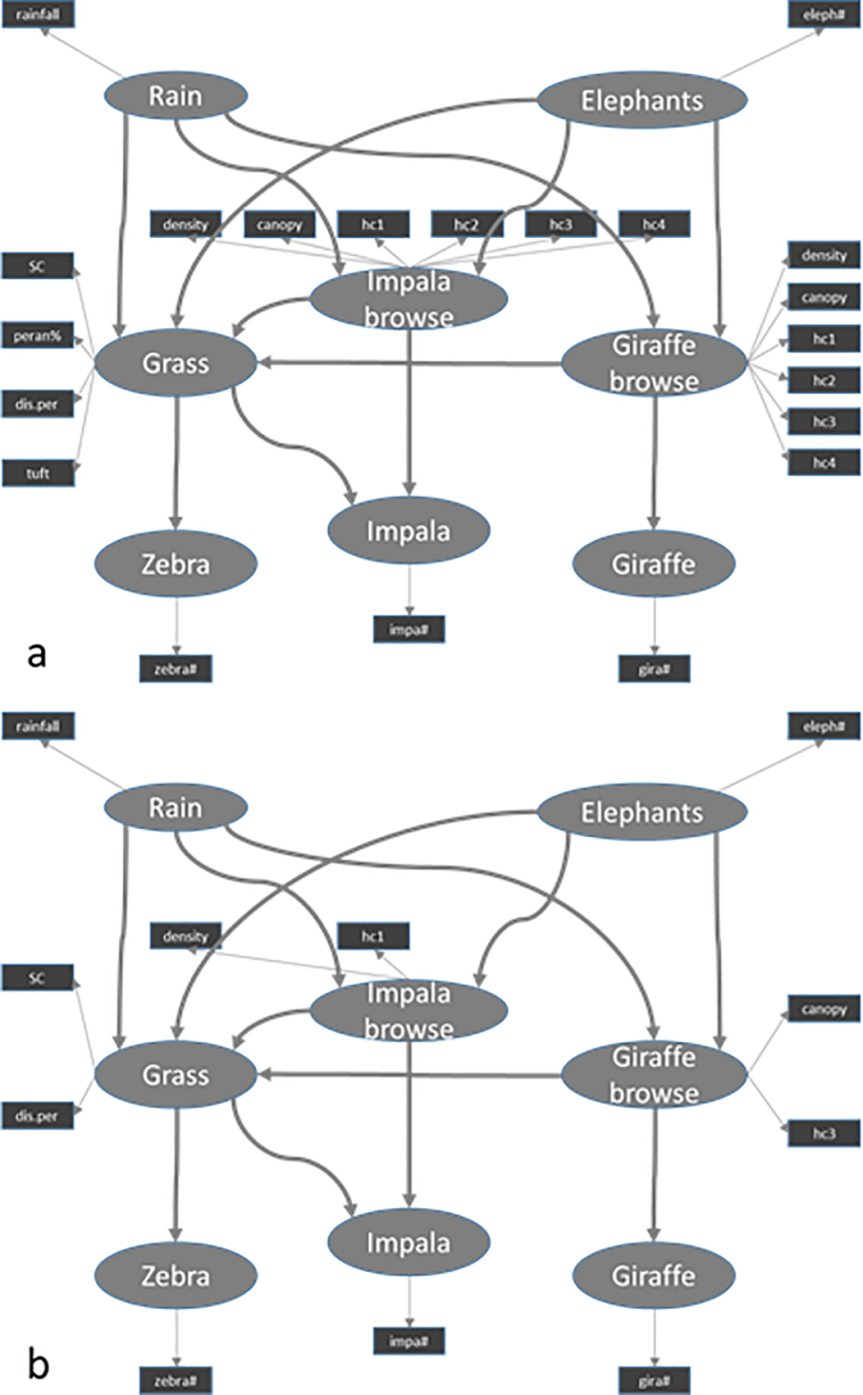
Initial total path model and used total path model. (a) The initial path model with all manifest variables which could describe the latent variables. (b) The final path model after removing manifest variables. Goodness of fit = 0.49. Circles show the latent variables and squares show the manifest variables. Arrows between latent variables indicate interactions and arrows toward manifest variables show which variables describe the latent variable.

For the outer model a selection had to be made of the possible manifest variables that best describe the latent variables of the inner model. A path model was therefore constructed using all logical manifest variables for all latent variables (Fig 1A). After running this model, each manifest variable was checked if it was a good descriptor on the basis of their loadings and weights (Sanchez 2013). If their loading was less than 0.7, which is considered acceptable in path modelling, they were removed from the analysis. If all descriptors were satisfactory, the integrity of all latent variables was checked with the Crombach alpha, the Dillon Goldstein’s rho and the first and second eigenvalue [22]. If these were all satisfactory, the variables were included as manifest variable (Fig 1B).

**Fig 2:**
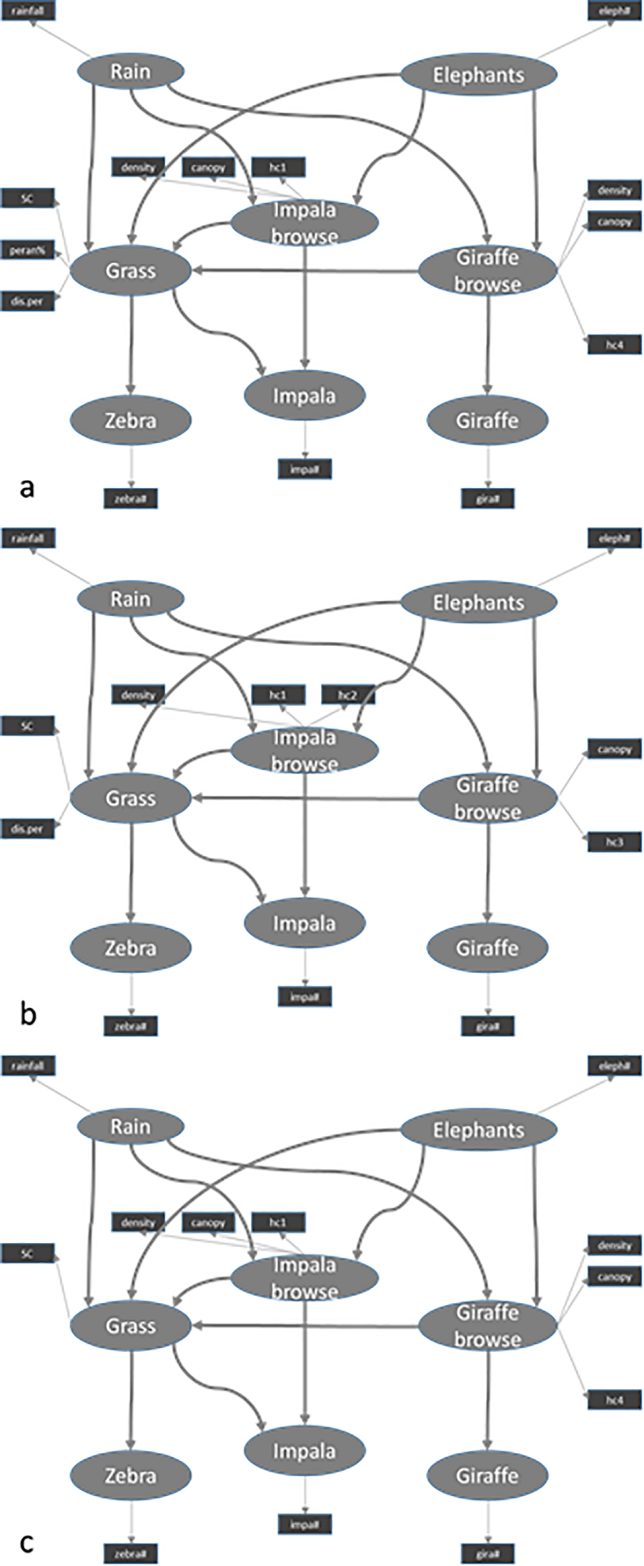
The final path models. The final path model for (a) Balule Game Reserve (Goodness of fit = 0.56), (b) Kapama Game Reserve (Goodness of fit = 0.64) and (c) Sabi Sand Wildtuin (Goodness of fit = 0.64) after removing bad manifest variables. Circles show latent variables and squares manifest variables. Arrows between latent variables indicate correlations and arrows toward manifest variables show which variables describe the latent variable.

We constructed a single path model, pooling all three areas, and a separate path model for each of the three areas, as interaction might differ between areas, under influence of e.g., differences in elephant densities or environmental factors. The interaction diagrams for the separate analysis was the same as for the overall analysis, except for some of the manifest variables, i.e. the descriptors of the latent variables (Fig 2A–2C).

## Results

**Fig 3:**
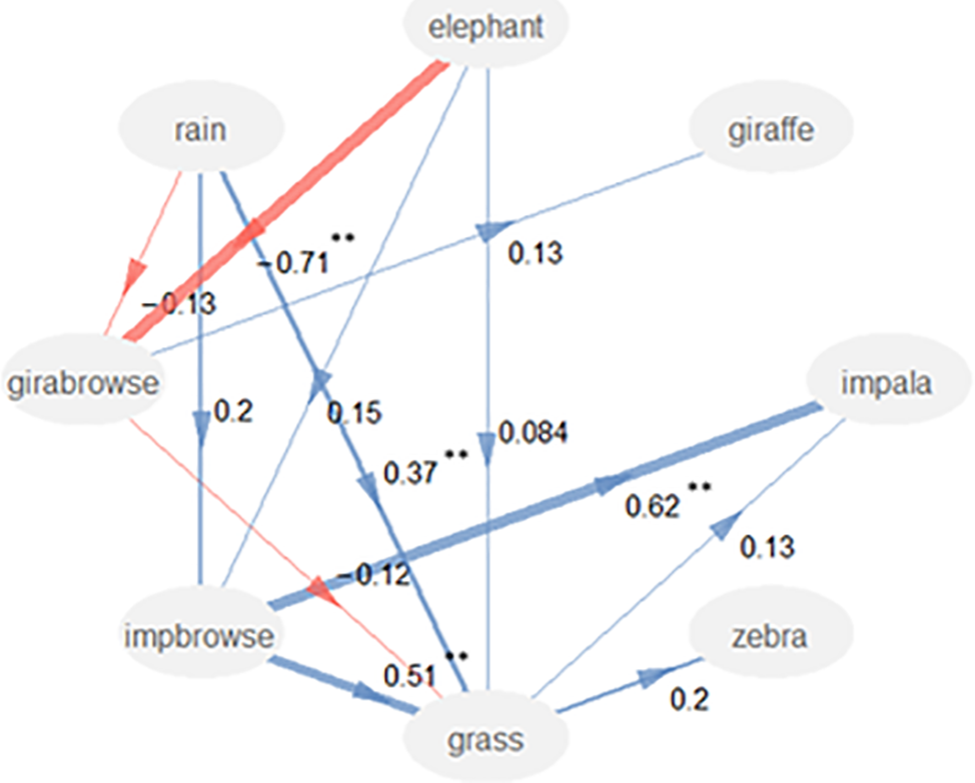
Results of the path model for three areas pooled together. Circles are latent variables and arrows illustrate interactions. Numbers are regression coefficients. * = P<0.05, ** = p<0.01. Girabrowse is browse availability for giraffes and impbrowse is browse availability for impala.

When the data of all three reserves were pooled (Fig 3), there was a significant negative correlation between elephant density and giraffe browse availability included in the path model. This correlation between elephant densities and impala browse and grass availability was not selected. Rain had a significant correlation with grass availability, but not with giraffe and impala browse availability. Surprisingly, there was no significant correlation between giraffe density and giraffe browse availability, and not between zebra and grass availability. For the impala, there was a significant correlation with impala browse availability, but not with grass availability. The expected competition between browse and grass was not significant, and there was even a significant positive correlation between impala browse availability and grass availability.

**Fig 4:**
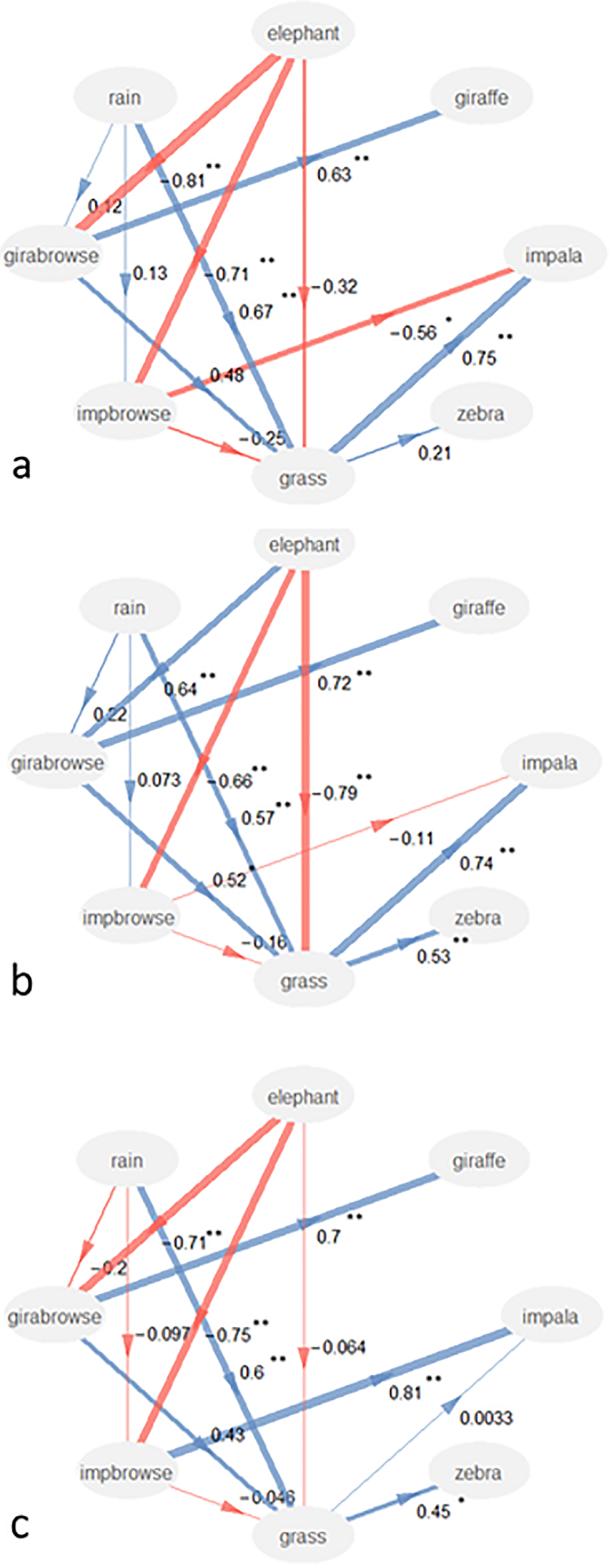
Results of the path models per reserve. Results of the path model for (a) Balule Game Reserve, (b) Kapama Game Reserve and (c) Sabi Sand Wildtuin. Circles are latent variables and arrows interactions. Numbers are regression coefficients. * = P<0.05, ** = p<0.01. Girabrowse is browse availability for giraffes and impbrowse is browse availability for impala.

In Balule Game Reserve (Fig 4A) there was a significant negative correlation between elephant density and impala browse availability, between elephant density and giraffe browse availability, and between impala browse availability and impala density, but no significant correlation was found between elephants and grass. There was a significant positive correlation between rain and grass availability, giraffe browse availability and giraffe density, and between grass availability and impala density. The expected competition effect between grass and browse was not selected. The correlation between zebra and grass availability was not significant.

In Kapama Game Reserve (Fig 4B) the negative correlation between elephant density and grass was significant, and there was again a significant negative correlation between elephant density and impala browse availability. There was a significant positive correlation between giraffe browse availability and giraffe density, grass availability and zebra density, grass availability and impala density, and between rain and grass availability. As opposed to Balule, there was also a positive correlation between elephant density and giraffe browse availability. There was a positive correlation between grass and giraffe browse.

In the Sabi Sand Wildtuin (Fig 4C) there was a significant negative correlation between elephant density and giraffe, and with impala browse availability. There was a significant positive correlation between rain and grass availability, giraffe browse availability and giraffe density, and between grass availability and zebra density. Again no competition effect between grass and browse was found. There was no significant impact of elephants on grass and Sabi Sand was the only protected area where impala was significantly correlated with browse availability, but not with grass availability.

## Discussion

The path models showed many interesting correlations. Rain had a significant positive correlation with grass, but not with browse availability. This is probably due to the fact that trees are able to reach the soil water while the roots of the grass do not reach it [7]. Therefore when there is less rainfall, the grass layer decreases, but the trees could use soil water for a long period of time. There was a strong negative correlation between elephant density and tall trees (giraffe browse availability), which was in line with our prediction, but no significant correlation with small trees or grass availability. Therefore it seems that elephants do indeed debark, and push over taller trees in order to get access to the leaves, which are otherwise unreachable, and in the process they decrease the tall tree density.

A surprising result was that there was no significant correlation between giraffe browse availability and giraffe density, and between grass availability and zebra density. A possible explanation for this can be that the browse and grass availability was not limiting for giraffe and zebra, but this could be different in the separate reserves. Impala numbers correlated stronger with browse than with grass. This is against the expectations, since impala prefer to feed on grass when this is available [8]. Possibly, this can be explained again by the grass not being limiting for impala, there might have been enough grass to feed impala, even in relatively poor years.

The expected competition effect between browse and grass was not detected in the overall path model. There even was a significant positive correlation between impala browse availability and grass availability. However, it is possible that this correlation is caused by a third variable, because there was both a positive effect of rain on grass availability and of rain on impala browse availability, so that a positive correlation was detected between grass and impala browse availability without any facilitative effects.

The results from the three separate path models showed that there were indeed different interactions in different areas, probably under influence of differences in management and/or environmental factors. The correlations with rain were quite similar to the overall path model for all three areas, but there were some clear differences in interactions between elephant densities and vegetation variables. Where there was no significant correlation in the overall path model, there was always a strong negative correlation between elephant density and impala browse availability in each of the three areas. There was also always a negative correlation between elephant density and grass availability, although this was only significant in Kapama. This suggests that the differences in elephant densities compared to browse and grass availability in the three areas can be so large that some correlations were not selected in the overall path model, even when these correlations were detected in all separate areas. These large differences in correlations suggest that resources are not always limiting. For example in Kapama there was a strong negative correlation between elephants and grass, while in Sabi Sand this correlation was not significant. Sabi Sand had the highest rainfall of all reserves and had a high grass availability compared to Kapama with average rainfall. This suggests that the grass is not limiting elephants in Sabi Sand and hence no strong correlation was found. In Kapama the grass seems to be limiting and the grazing pressure of elephants was strong enough to have an impact. In Balule there was however also a weaker correlation between elephant density and grass availability, while this area had the lowest average rainfall. This can maybe be explained by the fact that elephants can choose whether to browse or to graze. Elephants prefer to graze when there is plenty of grass, but change their diet when grass becomes scarce [8]. This might be the case in Balule. An alternative explanation for the different results in Balule and Sabi Sand compared to Kapama, is that Kapama is the only reserve of the three which has no open borders to Kruger National Park. Therefore in the dry season, the elephants are able to migrate to areas with a higher grass availability if there are open borders to Kruger, while in Kapama they have to stay in that area and feed on grass all year round. This can result in a stronger negative correlation between elephants and grass availability.

These results also indicate that studying the impact of elephants on vegetation cannot be done in one area only. A recent study done in the Sabi Sand Wildtuin showed that elephants have a stronger negative impact on browse than on grass, and that mesobrowsers are not able to benefit from the facilitative effects of elephants pushing over trees, and that therefore the herbivore community shifted to a grazer dominated community [11]. This was confirmed by the path model for Sabi Sand, but in an area with lower rainfall such as Balule and Kapama, the impact and cascading effects of elephant densities are very different. A possible interpretation is that elephants have a heavier impact on trees and larger cascading effect on other herbivores when there is lower average rainfall, which is in contradiction to the findings of Fritz *et al*. [4].

An overall trend was the positive correlation between giraffe browse availability and giraffe density. This suggests that giraffe always depend on browse availability and that there was no surplus of browse in these three areas. There was also always a positive correlation between zebra density and grass availability. However, in some of the reserves the grass did not seem to be limiting and the correlation was less strong. For example, in Sabi Sand the correlation was weaker than in Kapama, possibly because Kapama had lower rainfall and therefore the grass could have been limiting. This suggests that, as browsers could be affected by browse, and grazers by grass availability, together with the observation that grass had a stronger correlation with rain, grazers could be more affected by drought than browsers, which corresponds with findings from a previous study [6]. Additionally, the correlation between grass and zebra densities was strongest in Kapama. This could be because, without access to Kruger National Park, zebra might be more dependent on the local grass availability in the conservation area itself.

The expectations were to find a stronger competition effect between impala browse availability and grass than between giraffe browse availability and grass because of niche differentiation. In all three areas there was, however, a positive correlation between giraffe browse and grass, and a negative correlation between impala browse and grass. None of these correlations was significant, but the strength of the correlations differed between areas. A possible explanation could be the niche differentiation between large trees and grass. There will not be competition for the same resources because of different root systems, but good rainfall would have a positive impact on both large trees and grass. Moreover, tree canopies can improve the overall quality of the sub-canopy vegetation during wet season by improving nutrient uptake [23], and a larger canopy can provide shade for palatable, shade loving grass species such as *Panicum maximum* [24], resulting in a positive correlation between these two variables. A possible expansion of the model could include seasonality, since seasonal trade-offs exist between facilitation and competition [3]. Moreover, the positive effect of rain on both grass and trees might hide part of the competition effect.

Mixed feeders such as impala prefer grass over browse [8,10]. This was supported by the separate path models. At low and average rainfall (Kapama and Balule) there was a positive correlation between grass and impala density and a negative correlation between browse availability and impala density. The positive correlation suggests that grass can be limiting for impala. The negative correlation with browse seemed less logical, but a possible explanation could be a grass-browse competition effect. However, an increase in impala browse would then have a negative impact on the grass availability, and because impala feeds mostly on grass, a negative correlation between impala browse and impala numbers could arise. In Sabi Sand there was no correlation between grass availability and impala density, suggesting that grass was not limiting for impala just as it was not limiting for elephants. The expectation was that impala preferred grass over browse. This expectations seems to be supported by the separate path models, even though it did not show clearly in the overall analysis in which all reserves were combined.

This study provides us with new possible explanations for interactions and cascading effects. However, it is still a correlative and approximate study with a relatively low sample size and the results and reported correlations cannot be taken as causal relationships. The different correlations provide us with new insights in the dynamics of savanna ecosystems and indicates that more experimental research should be done to properly answer questions with regard to causal relationships. The path models provide us with a useful tool for visualizing possible interactions in savanna ecosystems. Provided a conservation area has an extensive dataset on animal numbers and available vegetation and rainfall data, a manager can use path models to predict the direct and cascading effects of, among others, an increasing elephant population. The common perception of managers in South Africa is that high elephant densities are destructive for the system, while this has not been proven in most of the cases. By using a path model, it can be estimated what effect the growth of an elephant population has for a specific conservation area. With this information, expensive and definitive process of culling or immunocontraception can be avoided if the impacts of current elephant density are different from expectations.

In order to make predictions more precise, more factors should be included, such as fire. The frequency and intensity of fires is largely determined by the fuel load [25]. Fuel load is higher if there are more grasses, increasing the fire frequency. Fires can be destructive towards trees, since they take much longer to regrow then grass. Therefore through competition, a higher frequency of fire could increase the grass availability. A higher grass availability often means a higher frequency of fires, which could trigger a positive feedback loop [26]. Elephants can open up a landscape and thereby increase fire frequencies. This means that they impact large trees, and through competition release, grass availability increases [5]. Therefore higher elephant densities are expected to be correlated with a higher fuel load and a higher frequency of fires. This way elephants and fires can reinforce each other’s effects on grass and browse availability, changing the interactions between elephants and other savanna ecosystem components. Since controlled fires is also one of the most commonly used management tactics to open up the landscape, it is important to understand the direct as well as the indirect effects of these interactions. It is important to incorporate this into the analysis, since it has already been shown that the effects of fires can differ between areas, and that cascading effects are not fully understood [26].

Predation is another interesting topic to include in the path model. The impact of predation on herbivore species is size-dependent. Smaller predators can only feed on the smaller prey animals, while larger predators can feed both on larger herbivores, small herbivores and even on the smaller predators, while megaherbivores, such as the elephant and rhinos, are generally not predated at all [27]. Predation size effects can shift a herbivore community. Moreover, a growing elephant population opens up the landscape, affecting the hunting success of predators. Different effects of an open landscape have been suggested for different species of predators and prey. Larger herbivores such as giraffe and kudu may benefit from an open landscape, because of increased visibility and predator detection [2]. Smaller herbivores, such as bushbuck, may be negatively affected by an open landscape, because the thicker vegetation supplies them with refuge against large predators [28]. So, extending the path model with predators and including top-down effects that are mediated by vegetation composition, and bush cover, influencing the predation risk of large and smaller herbivore species, is a promising addition to the model.

We conclude that interactions in a savanna ecosystem are dependent on various factors and impossible to analyse with direct one-by-one correlations. PLS-PM is good tool to visualize the different correlations in a single diagram [22]. Furthermore, due to the complexity of the ecosystem and the effect of management and environmental factors, processes should not be studied with data of multiple areas pooled together, but separate path models are required, distinguishing patterns in separate areas. This way, also co-varying variables, in reaction to a third variable, can be better studied, the difference between correlation and causation becomes clearer [29], and interactive effects with e.g. local conditions in rainfall or animal movements can be studied better. Path modelling can be a useful tool for visualizing possible direct, indirect and cascading effects of changing animal densities in specific areas.

## Supporting information

S1 Table Data file with all manifest variables per reserve per year. (XLSX)
